# Magnesium and Hypertension: Decoding Novel Anti-hypertensives

**DOI:** 10.7759/cureus.25839

**Published:** 2022-06-10

**Authors:** Nassar Patni, Mahejabeen Fatima, Aselah Lamis, Shiza W Siddiqui, Tejaswini Ashok, Ahmad Muhammad

**Affiliations:** 1 Internal Medicine, Deccan College of Medical Sciences, Hyderabad, IND; 2 Research, Dubai Medical College, Dubai, ARE; 3 Internal Medicine, Jagadguru Sri Shivarathreeshwara (JSS) Medical College, Mysore, IND; 4 Cardiology, Deccan College of Medical Sciences, Hyderabad, IND

**Keywords:** anti hypertensive, magnesium, hypertensive heart disease, cardio vascular disease, magnesium deficiency, hypertension

## Abstract

Hypertension (HTN) is a complex multifactorial disease that is one of the most prevalent disorders in our modern world. It can lead to fatal complications like coronary artery disease (CAD) and congestive heart failure (CHF) in high-risk individuals. The silent nature of HTN also contributes to its immense caseload and, today, with a number of combinations and various antihypertensive agents, patient compliance is becoming increasingly difficult. This article has reviewed the role and mechanisms of magnesium (Mg) in reducing HTN in the human body so as to provide more information that may help include it as a mainstream antihypertensive regimen. This review has also shed light on the cardioprotective nature of Mg against pathologies like CHF with special mention to patient groups who are at high risk for low Mg levels. Many studies included in this article solidify the former link, but some also provide contradicting data.

## Introduction and background

Hypertension (HTN) is diagnosed when an individual’s systemic arterial blood pressure (BP) is above a threshold [1]. Today worldwide, this threshold has been established at BP above 140/90 mm of mercury (Hg) [1], although a more inclusive view is needed as HTN-related cardiovascular disease (CVD), a complex association of chronic HTN, presents at BP values different from those used for HTN diagnosing thresholds mentioned earlier [1]. The concerns about chronic HTN began when risks of elevated BP started to be documented in various epidemiological studies, beginning with the Framingham Heart Study in 1948; such studies continue to the present day [2-5]. In 2005, Kearney et al. estimated that HTN is expected to increase to a total of 1·56 billion (1·54-1·58 billion) affected adults worldwide by 2025 [6].

Moreover, increasing age profoundly influences risk factors for developing HTN. In an analysis of a million individuals in 61 epidemiological studies followed for 13.3 years, the risk factor for CVD in individuals with a higher BP was the same as in individuals who were 20 years older but had a lower BP [7]. HTN has always been prevalent in ethical/racial minorities and thus they have a higher risk of HTN-related morbidity and mortality [8]. In a study conducted in the United States (US), African Americans had the highest prevalence of HTN, at 56%, followed by Hispanics at 34% [9-11]. However, systemic racial discrimination and substandard medical care could be contributing factors.

HTN has complex pathophysiology with many factors at play. Hence, it is best to take a mosaic approach inclusive of all aspects and systems; Irvine Page first proposed this in 1949 [12]. However, cardiac and vasculature systems are the key modulators responsible for most changes in one’s BP [13]. HTN has been primarily viewed as an asymptomatic disease; however, chronically elevated levels present with several complaints. A descriptive cohort study on 266 individuals with chronic HTN showed that 83.83% had fatigue, 73.68% had body aches, 46.99% had headaches, 36.84% had shortness of breath, and 9.40% with nasal bleeds [14]. There is enough evidence-based information to deploy more than 100 antihypertensive medications and more than 50 combination products to reduce HTN-associated mortality and morbidity [12]. One meta-analysis involving 41 clinical trials involving 137,260 patients compared an active antihypertensive agent with a placebo or no treatment. There was a significant reduction in all-cause mortality in patients on an active antihypertensive agent [15]. This proves the efficacy of deploying antihypertensive agents.

Presently, HTN has a considerable burden on patient caseload worldwide. Its association with fatal CVD urges us to look into newer and better treatment options. In recent years, the importance of Mg in the human body has drawn the attention of all. It started when research linked low Mg levels to various pathologies like migraine, arrhythmia, diabetes mellitus, and dyslipidemia among others. However, the bulk of the research focused on the cardiovascular systems (CVS), and its physiological influence on the vascular system prompted numerous studies in HTN, despite mixed results. This article aims to review the mechanisms of Mg with some evidence-based studies to highlight its role in controlling HTN and to discuss whether it could be deployed in the mainstream antihypertensive treatment regimes.

## Review

Magnesium in human biology

After potassium (K), Mg is the second most abundant cation in the human body and serves as a cofactor for 325 enzyme systems, including specific vasoactive mediators [16]. Despite being abundant, Mg is mostly found intracellular, hence, serum levels might not be the best indicators of Mg stores in the human body. However, to talk numbers, a serum level range of 0.76 to 1.15 moles/liter is the average level [17]. To maintain this level, an average adult human’s recommended dietary allowance (RDA) requires about 420 milligram/day of Mg intake [18]. Humans get most of their Mg from simple everyday food items like green leafy vegetables, water, cereals, nuts, and legumes. However, with age, the absorption of Mg is reduced while the excretion from kidneys via urine is increased [19]. Magnesium deficiency (MgD) is mainly related to a lower intake of Mg than the RDA. Low food and water intake, including purified salt to cook or refined cereals and pulses, might result in a negative baseline over time.

**Figure 1:**
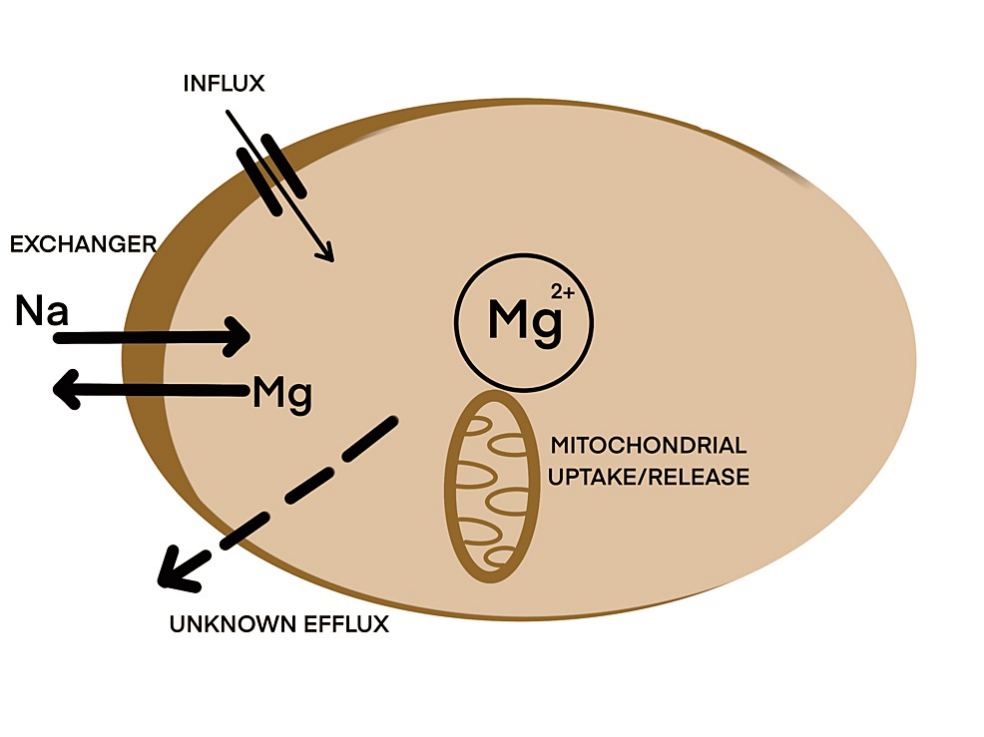
Homeostasis of magnesium in a vascular smooth muscle cell. Image credit: Nassar Patni Mg: magnesium; Na: sodium

To understand the mechanisms that have made Mg such a topic of discussion, one needs to understand the homeostasis of Mg and where in the body it has the most influence. Mg is not a static ion and, like many charged ions, it moves across the membrane and maintains the required balance between important cells like the vascular smooth muscle cells (VSMCs) and the cardiac cells [20]. Overall, Mg enters the VSMCs via Mg channels, with an active uptake/release by the cell’s mitochondria and sarcoplasmic reticulum (SR) [20,21]. Simultaneously, a sodium (Na)-Mg exchanger exists where Mg is exchanged for Na. Other components include an Mg buffer system that maintains homeostasis and an efflux channel about which not much is known [20,21]. These systems are not very well understood and need a more cellular and molecular level of research to learn about their constitution and properties. This is compiled into Figure 1, which shows all the influx/efflux mechanisms at play.

Antihypertensive mechanisms

Mg has various properties that have made it ideal for treating HTN. In general, intracellular Mg modulates VSMC tone while extracellular Mg influences calcium (Ca) channels. In a dissolved state, Mg has a tighter hydration bond than the Ca ion; hence, it’s difficult for it to undergo dehydration. These hydrated Mg ions act as natural Ca antagonists owing to their larger size. The second crucial function is its balance between VSMC-endothelial cells-vasoactive mediators. Naturally, these also happen to be systems with the most significant influence on a human’s BP.

1.1 Calcium Antagonist

To understand why and how this occurs, we need a more cellular level of understanding. A hydrated Mg ion is far different from its non-hydrated counterpart, and its radius is about 400 times larger than a dehydrated ion. This property is the basis of most of the antagonist properties Mg possesses despite having a similar charge and reactivity to Ca [22]. The presence of Ca in the VSMC cytosol is one of the main principal determinants of its contractile property. Extracellular Mg inhibits Ca function inside the VSMC by two main mechanisms. Firstly, it neutralizes the negative charge of the plasma membrane of a VSMC cell, making it more stable and raising the charging threshold of the entire cell, and diminishing current via voltage-gated Ca channels [23]. Secondly, it directly binds to the Ca channel either by mechanically blocking it or allosterically modulating it [23]. Overall, Ca influx is terminated, and the efflux is reduced, making way for unopposed vasodilation. A third minor mechanism works intracellularly, where Mg inhibits the inositol-triphosphate 3 (IP3)-induced Ca release from the SR, reducing these vasoconstriction modulators [23]. Naturally, an Mg deficient state will cause IP3 mobilization of Ca from SR to reduce Ca adenosine triphosphatase (ATPase) activity that reduces Ca efflux, leading to vasoconstriction [24].

1.2 Vascular And Endothelium Mechanisms

**Figure 2:**
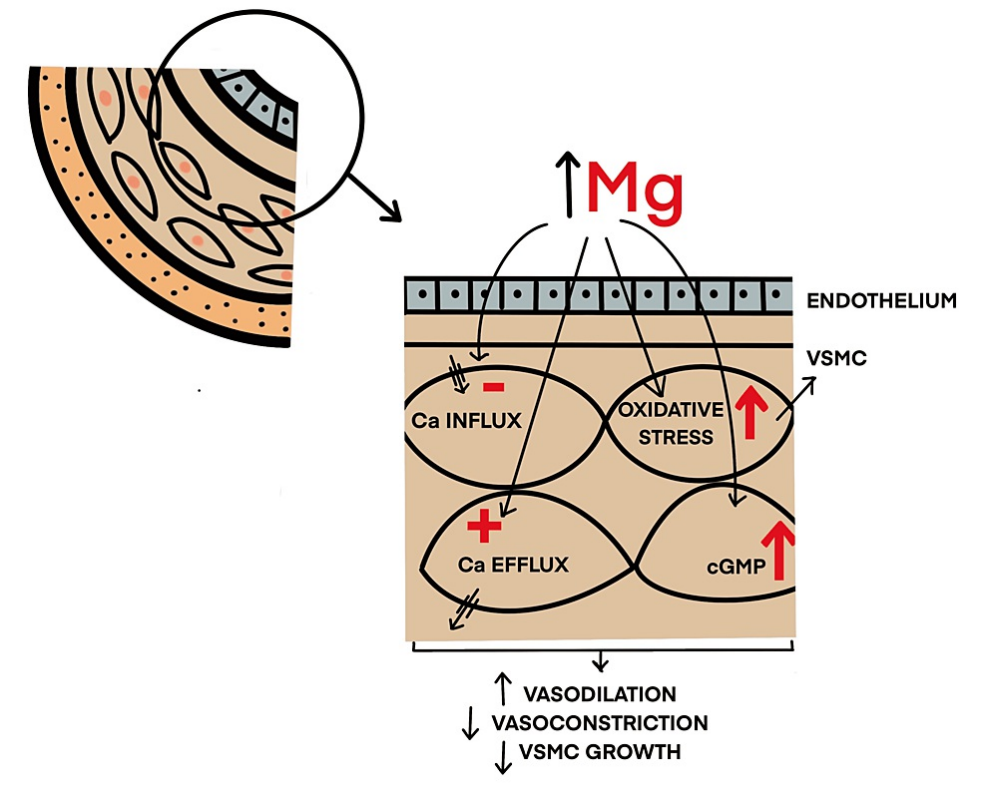
Anti-hypertensive action of magnesium via the vascular smooth muscle cells. Image credit: Nassar Patni Mg: magnesium; VSMC: vascular smooth muscle cell; Ca: calcium; cGMP: cyclic guanosine monophosphate

As stated, the vascular system is the most significant influencer in HTN, and now studies show Mg influences the vascular tone. In 1925, Blackfan and Hamilton first observed that Mg salt infusion lowered human BP by vasodilatory effect [25]. When looking at vascular reactivity of Mg, an increased extracellular Mg concentration is seen to improve blood flow, decrease vascular resistance, and increase the capacitance function of the peripheral, renal, coronary, and cerebral blood vessels. In contrast, a lower concentration will have opposite effects [26- 31]. Mg has an impact on the VSMC and on modulating endothelial function. A summary of this effect is showcased in Figure 2. The vascular endothelium significantly impacts the vascular tone and, invariably, the mean arterial pressure (MAP) in a human body. In experiments done on arteries of the human forearm, Mg caused endothelium-mediated vasodilation [32]. These actions are thought to happen due to the mediation of nitric oxide (NO), cyclic guanosine monophosphate (cGMP), and cyclo-oxygenase systems, all of which are potent vasodilators in the human body [33,34]. Yang et al. experimented on rat aortas and concurred with Mg concentration-dependent induced vasodilation and endothelium-independent relaxations on the vasculature [34]. Two more inferences to consolidate this idea were that the Mg producing effect was more profound on intact endothelial cells and less so on endothelial denuded cells in the experiments. This effect was inhibited when NO inhibitors were added. Showcasing the importance of both an intact endothelium and NO-mediated vasodilation [33,34].

1.3 Magnesium And Renin Angiotensin Aldosterone System

The renin-angiotensin-aldosterone system (RAAS) is a well-studied hormonal system of the human body. Angiotensin-2 (AT-2) effects include vasoconstriction via the AT-2 receptors on the microvasculature, increasing Na and water reabsorption via the proximal convoluted tubules (PCT) in the kidney, and releasing aldosterone that, in turn, acts on principal cells of the nephron for water, Na reabsorption, and K secretion. Rats fed a low Mg diet were observed to have proliferating cells in the zona glomerulosa of the adrenal cortex, which is the source of aldosterone hormone [35]. In addition, a higher juxtaglomerular granulation index was observed, indicating a higher overall renin release, while the inner zones of the adrenal cortex were slightly reduced [35]. However, in Mg recovering rats, the juxtaglomerular index and the width of zona glomerulosa both returned to normal [35]. This prompted the role of Mg in the RAAS of the human body, or essentially what the far-reaching role of Mg is on other hormonal systems of the human body. Although much research is needed In humans, it can be pointed out that individuals with HTN, with higher plasma renin activity, have deficient Mg levels [36]. The Ca antagonist property of Mg continues to be its most important factor having a reach beyond the VSMC. Mg in humans decreases aldosterone production by inhibiting the cellular Ca influx in the zona glomerulosa, a Ca-dependent process [35]. Finally, it was proven that MgD rats had increased plasma renin activity, aldosterone, and corticosterone levels. The authors also indicated they decreased AT-2 and aldosterone secretion on Mg supplementation in these rats [36].

1.4 Other Mechanisms

As stated in the introduction, the pathology of HTN is mosaic with several contributing factors. While reviewing the role of Mg, we also discovered the various hypothetical etiopathogenesis of HTN in humans. Chronic MgD leads to dysfunctional regulation in vascular and endothelial adaptive immune responses, contributing to HTN as it increases arterial stiffness [37]. Relatively new data that surfaced has added various concepts to the pathogenesis of HTN and linked low Mg with it. Chronic inflammatory and oxidative stress causes vascular and endothelial dysfunction that ultimately causes HTN [38]. The evidence is provided by animal studies that link HTN with both humoral and cellular immunity, particularly with low-grade vascular inflammation [38]. Studies show that the vascular stress increases as MgD initiates the production of pro-inflammatory molecules like interleukin (IL) 1, IL 6, tumor necrosis factor, vascular cell adhesion molecule, and plasminogen activator inhibitor-1 (PAI-1) and decreases antioxidant activity by reducing enzymes like glutathione peroxidase, superoxide di mutase, and catalase [36]. Other important antioxidants like vitamin C, vitamin E, and selenium are reduced [36]. Besides directly affecting vasoactive substances and VSMC, Mg also influences the sympathetic nervous chain that initiates this vasoconstrictive response. Evidence shows that Mg infusion in the rabbit peri arterial system reduces the sympathetic response initially triggered with Ca’s help via catecholamines [39]. The direct relation of Ca with catecholamine is well known, and Mg antagonizes this effect by acting on adrenal chromaffin cells and reducing acetylcholine (Ach) [40]. Another vital hypothesis is related to adenylate cyclases (ADCY), which are essential for their catalytic action on Ach on preganglionic nerves, which is necessary for keeping catecholamines from the adrenal gland in check, and Mg is required for this catalytic function of ADCY [41].

**Figure 3:**
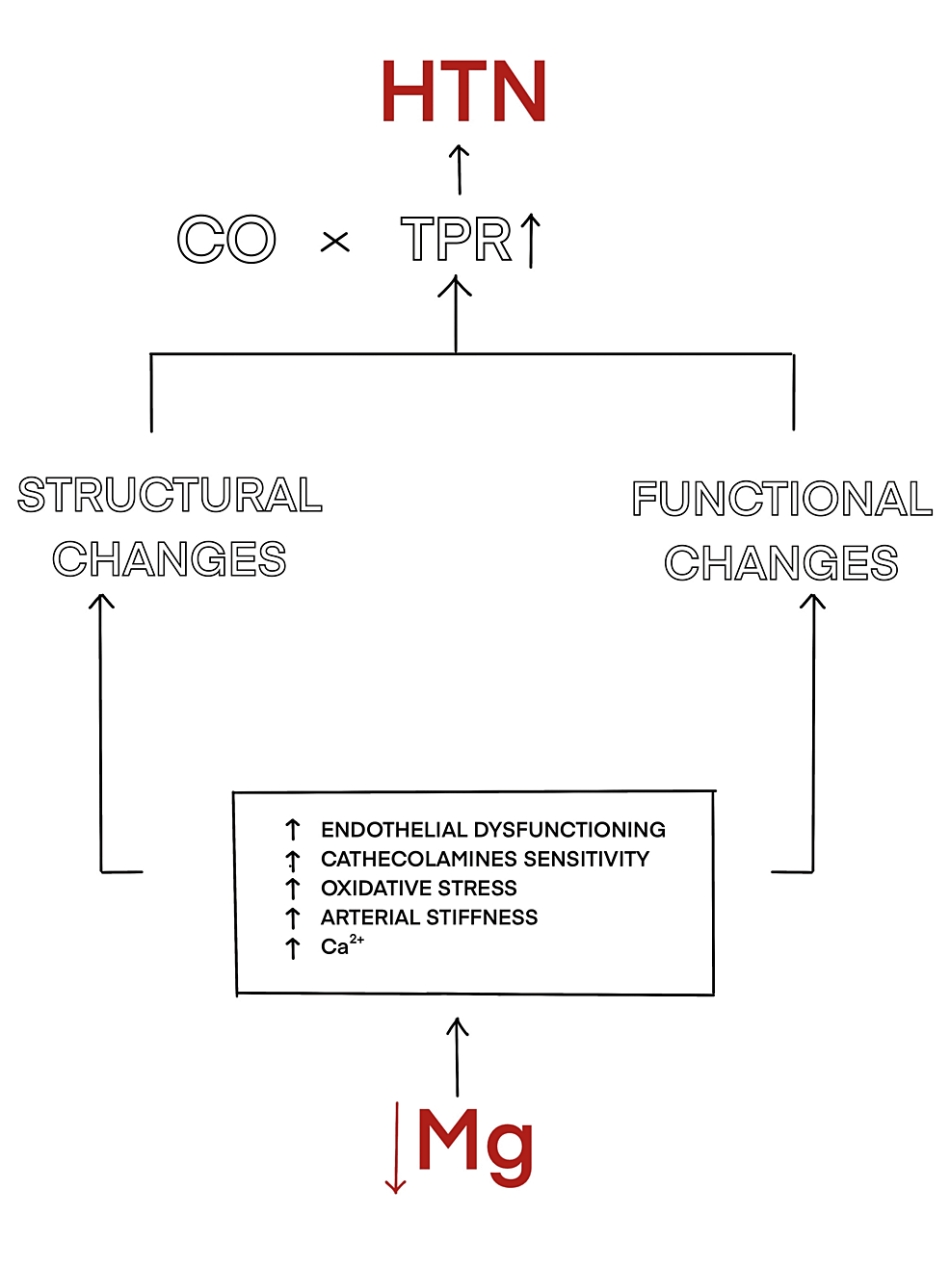
Structural and functional alterations in blood vessels from low magnesium levels, leading to hypertension. HTN: hypertension; CO: cardiac output; TPR: total peripheral resistance; Ca: calcium; Mg: magnesium

As shown in Figure 3, all of the above mechanisms provide a disastrous combination of structural and functional changes in a macro view. Structurally, the lumen of the artery is reduced, there is arterial stiffness, the lumen ratio gets narrower, and functionally due to vascular-endothelium dysfunctioning, suppression of vasodilation, and unopposed vasoconstriction leads to increased total peripheral resistance (TPR) and then ultimately raises the BP.

Epidemiological studies

Many animal studies linked Mg and other nutrients to lowering HTN, but studies on human beings were limited. Starting with a broader role of Mg by pitting it against other nutrient variables of the human diet, Joffres et al. published a study in 1987 where 615 men of Japanese origin in Hawaii were studied, and information on the nutritional variables in their diet was incurred from a healthy diet fact questionnaire. After reviewing the data, it was concluded that Mg had the most substantial inverse relationship with BP (Table 1) [42]. Among the earlier studies was a prospective cohort study conducted by Ascherio et al. in 1998; 43,738 men from the US with no previous diagnosis of CVD and HTN were taken as subjects. A semi qualitative food frequency form was given with consent, and subjects were followed for eight years. Although 328 stroke events were documented, 52 cases of stroke were in men with a rich Mg diet versus 74 stroke cases in their low Mg level diet counterparts. Moreover, an inverse relation was established between Mg intake and the risk of stroke (Table 1) [43].

More significant evidence came from a prospective cohort study by Kawano et al., where 60 human subjects with diagnosed HTN above 140/90 mm of Hg were studied in two phases. There was a control period of eight weeks where no intervention was given, followed by a supplementation period of eight weeks. Mg oxide of 20 millimoles (mmol) per day was given to each subject. Ambulatory, home, and office readings were noted for all issues during this period. The inference showcased a fall in office BP by 3.7⁄1.7 mm Hg, 24-hour ambulatory BP fell by 2.5 ⁄ 1.4 mm Hg, and home BP measurements by 2.0⁄1.4 mm Hg, further consolidating the role of Mg in HTN (Table 1) [44].

**Table 1:** Summary of studies showing role of magnesium in reducing blood pressure. h/o: history of; HTN: hypertension; CVD: cardiovascular disease; Mg: magnesium; US: United States; RR: relative risk; BP: blood pressure; SBP: systolic blood pressure; DBP: diastolic blood pressure; mmol: millimoles; OPD: outpatient department; K: potassium; Ca: calcium; RCT: randomized controlled trial; hg: mercury

REFERENCE STUDY	STUDY DESIGN	SUBJECTS	METHODS	RESULTS
Joffres et al., [42]	Prospective cohort	615 Japanese origin in Hawaii, born between 1900 and 1919.	61 nutritional variables studied in men with no h/o HTN or CVD.	Among all dietary variables, Mg had the strongest inverse relation to HTN.
Ascherio et al., [43]	Prospective cohort	43,738 US men between the age of 40 to 75 years.	The sample had no H/o HTN or CVD and was given a food frequency questionnaire in 1987 with regular follow-up.	Multivariate RR of stroke with higher Mg intake was 0.70(95% CI,0.49,1.01; P for trend <0.027).
Kawano et al., [44]	Prospective cohort	60 adults aged 34 to 75; 34 men and 26 women population	Population with SBP of more than 140/90 on two office occasions were given an eight-week control period followed by a supplementation period (mg oxide 20 mmol per day).	After supplementation, office BP fell by 3.7/1.7 mm Hg, 24h ambulatory by 2.5/1.4, and home BP by 2.0/1.4 mm Hg.
Witteman et al., [45]	Double-blinded control trial	91 middle-aged to older women from the Dutch town of Zoetermeer.	Women with mild to moderate HTN. Divided into a placebo group and supplemented group (mg aspartate HCL 20 mmol/day).	SBP falls 2.7 mm hg (95 % CI -1.2,6.7;P <0.18) DBP by 3.4 mm hg (1.3,5.6,P < 0.003).
Hatzistavri et al., [46]	Prospective cohort	48 Adults from HTN OP of Aristottle university of Tessaloniki.	24 were given 600mg Pindolate Mg + lifestyle changes. While others only lifestyle changes.	SBP – 5.6 +/-2.7 with P<0.001 . DBP -2.8 +/-1.8 P<0.002.
Patki et al., [47]	Double-blinded randomized placebo-controlled trial of 32 weeks	37 adults from the OPD of Sassoon General Hospital, Pune, India.	37 adults with HTN and DBP <110 mm hg gave either placebo or 60 mmol per day of potassium or potassium with 20 mmol per day of Mg.	K alone or K with Mg had equal reduction with P < 0.001.
Sacks et al., [48]	Clinical trial	135 adults, 82 men, and 43 females.	The population was given Ca, K, and Mg combinations of 60, 25, and 15 mmol per day.	A minimal difference in SBP and DBP (95% CI) was noted. -1.3(-4.4 to +1.8)/-0.4(-2.9 to +2.1) for K-Mg and +2.1(-1.8 to +6.0)/2.2(-1.0 to 5.4) for Mg -Ca.
Dickinson et al., [49]	Meta-analysis	Six RCT with 483 Adults over 18.	Inclusion criteria for trials were Mg supplementation on subjects, while controls were given placebo or no treatment.	Five RCTs showed no data to link low Mg with HTN (statistically insignificant), While one African-based RCT showed little inverse relation of significance.
Mizushima et al., [50]	Meta Analysis	Reported 30 separate analysis sets from 29 observational studies.	Varied methodology, 24-hour recall (n=12), food frequency questionnaire (8), food record (7), and duplicate diet (2).	Pearson r correlation for 13 subgroups was done, and eight showed no relation of Mg with HTN.

More promising data came from a double-blinded control trial conducted by Witteman et al. between 1985 and 1988 on 91 Dutch women followed up for six months. Subjects were diagnosed as women having mild to moderate HTN; they were divided into two groups. One group was supplemented by 20 mmol per day of Mg aspartate, and the other group was given a placebo. A significant systolic blood pressure (SBP) reduction of 2.7 mm Hg (95%CI -1.2, 6.7; P = 0.18) and diastolic blood pressure (DBP) by 3.4 mm Hg (1.3, 5.6; P = 0.003) was noted (Table 1) [46]. Within the last two decades, a significant study by Hatzistavri et al. in 2009 was conducted on 48 patients with mild, uncomplicated HTN taken at the Aristotle University of Thessaloniki, Greece. Twenty-four of them were given a dose of 600 mg of magnesium pidolate per day with lifestyle changes, while 24 were just given lifestyle changes. Followed by 12 weeks, the overall SBP was reduced by 5.6 mm Hg and DBP by 2.8 mm Hg with p values of <0.001 and <0.002, respectively, indicating that the study was clinically significant (Table 1) [45]. Interestingly, giving a more neutral stance, a small study conducted by Patki et al. in Sassoon, India, showcased highly controversial data. A double-blinded randomized controlled crossover study of 32 weeks involving 37 adults with mild HTN concluded that K supplementation reduced BP with a statistically significant value of p<0.001. Adding Mg to this group has no additive effect in lowering HTN. Hence, K alone or K with Mg supplementation gave similar reductions with a P<0.001 (Table 1) [47].

Although the above studies and trials favor this review, there have also been many studies with contradicting data. In 1995, a clinical trial was conducted comprising 135 human subjects performed by Sacks et al. Participants were given combinations of three vital supplementations. These included 60 mmol per day of Ca, 25 mmol per day of K, and 15 mmol per day of Mg. The result showed no significant reduction in either Mg supplementation or in other combinations. The difference in SBP and DBP with 95% CI was -0.7 (-4.3 to +2.9) and -0.4 (-2.9 to +2.1) for K-Ca , -1.3 (-4.4 to +1.8) and 0.4 (-2.5 to +3.3) for K-Mg, and +2.1 (-1.8 to +6.0) and +2.2 (-1.0 to +5.4) for Ca-Mg combination (Table 1) [48]. Some of the strongest evidence of contradicting data comes from meta-analyses. One such published in 2003 by Dickinson et al., where six randomized control trials (RCTs) with up to 16 weeks of follow-up that met the inclusion criteria concluded that five trials did not yield statistically significant data showing the reduction in SBP (mean difference -11.2, 95%CI: -25.2 to 2.7) and DBP (mean difference: -5.0, 95%CI: -12.5 to 2.4). Excluding one trial on African population where it resulted in a small reduction in SBP, (SBP mean difference: -3.9, 95% CI: -8.6 to 0.8; DBP mean difference: -1.5, 95% CI: -6.2 to 3.1). However, pregnant women and patients on antihypertensive medication were a strong exclusion point (Table 1) [49]. Another was published by Mizushima et al. that included 29 observational studies. From these, 30 separate analysis sets were derived and studied. These trials varied in methodology. Information was procured by the 24-hour recall, a food frequency questionnaire, or food records. The Pearson- r correlation was reported after adjustments were made for confounding factors. Among them, eight showed a negative relation between SBP and DBP. Hence, no relation exists between Mg intake and HTN. The study further recommended further studies to establish a strong evidence-based link (Table 1) [50].

Magnesium and the CVS

HTN is an entity linked to various pathologies of the CVS. It is the most significant risk factor in life-threatening conditions like CAD, chronic heart failure (CHF), and atherosclerosis. An analysis of patients with chronic kidney diseases (CKD) and cardiovascular pathologies might help us integrate the link between Mg and HTN. Patients with chronic kidney conditions and regular diuretic usage are prone to low Mg levels via hypomagnesemia. Because the kidney is the principal regulator of Mg serum levels, it is essential to address these patients because low Mg levels will have a more significant impact on HTN and CVS if these systems are affected at all. The prevalence of MgD increases by 11% and 9.3% in hospitalized patients [51] and goes up to 65% in intensive care units [52]. Two extensive cohort studies on hemodialysis patients indicated a significant risk of cardiovascular death due to low Mg serum levels [53,54]. More clues can come when studying congenital Mg deficiencies syndromes like Bartter and Gitelman Syndrome. Mg wasting conditions produce cardiovascular disturbances like prolonged QT interval on echocardiogram (ECG), exercise-induced myocardial perfusion defects, ventricular tachycardia, and sudden cardiac death [55-59].

Although these studies are controversial, Mg supplementation might reverse these effects. The atherosclerosis risk in communities with low baseline dietary Mg intake contributed to the significant incidence of CAD by adjusting for other risk factors [60]. By mechanisms already stated earlier, low Mg leads to chronic vascular dysfunction, vasoconstriction, increased arterial stiffness, and higher oxidative stress and ultimately contributes to lumen narrowing, resulting in a coronary artery stenosis incident [36,37,38]. There has been much talk about the cardioprotective nature of Mg. Mg provides protection against myocardial ischemia by reducing intracellular Ca overload, conserving cellular ATP, reducing myocardial oxygen consumption, attenuating catecholamine-induced high oxygen demand, and protecting the post-ischemic myocardium from oxidative damage [60]. Mg might be more important in human bodies than we think. It has been implicated in the pathophysiology of heart failure because Mg is an essential cofactor in ATP synthesis in mitochondria and energy production in cardiac myocytes [61].

Moreover, intracellular Mg mobilizes Ca into the SR for excitation-contraction coupling [61]. Hence, it makes sense why it would be implicated with such a fatal outcome. A prospective cohort meta-analysis showed a 22% decrease in risk of CHF only with an increase in Mg in diet [62]. In class two and four CHF, administration of Mg decreases per mature ventricular depolarisation and increases mortality [63]. Although it must also be mentioned that the prospective protocol used management in sepsis (PROMISE) study of more than 100 patients with three and four CHF showed no correlation between Mg intake and decreased mortality or survival [64].

Limitations

This review aimed to highlight and establish an inverse link between Mg and HTN. HTN is a vast topic with its roots deep into many CVDs, and this review could not cover every relation of Mg-HTN-CVS. Two particular issues that were close to this review were not included. First is the pharmacology, pharmacokinetics, and adverse effects of oral/intravenous Mg supplementations and patient compliance. The second issue would be a lack of data on Mg’s general mortality and morbidity-lowering outcome, assuming any exists, as this is the ultimate goal in the hope to deploy it as a mainstream antihypertensive medication.

## Conclusions

The goal of this review was to provide a broad overview of the evidence for a negative relationship between magnesium levels and HTN. This inverse relation comes from various mechanisms of action, most noteworthy being the Ca antagonizing property, vascular and endothelium dysfunctioning, increased arterial stiffness, and Mg’s contribution to the RAAS system are other worthy mentions. A more cardioprotective nature of Mg was also discussed with evidence on preventing fatal sequels like CAD and CHF in humans. The contribution of this review, if clubbed with other clinical trials on Mg supplementation, would have a profound influence on reducing the burden of HTN and, hence, CAD in present times. Apart from reducing case loads, this review can contribute to making an established link that would help make population lifestyle modifications. More conscious consumption of Mg-rich food will be maintained, which would mean grassroot primary prevention. In medical practice, Mg supplementation could also reduce the need for several antihypertensive combinations that are currently deployed and would increase long-term patient compliance. Although some meta-analyses did showcase no established link for this inverse relation, we recommend further research, reviews, and more clinical setting trials on humans.
